# Development of computer-controlled atmospheric pressure plasma structuring for 2D/3D pattern on fused silica

**DOI:** 10.1038/s41598-021-01592-w

**Published:** 2021-11-17

**Authors:** Duo Li, Peng Ji, Yang Xu, Bo Wang, Zheng Qiao, Fei Ding

**Affiliations:** Center for Precision Engineering, Harbin Institute of Technology, Harbin, 150001 China

**Keywords:** Engineering, Optics and photonics

## Abstract

Fused silica with structured and continuous patterns is increasingly demanded in advanced imaging and illumination fields because of its excellent properties and functional performance. Atmospheric pressure plasma, based on pure chemical etching under atmospheric pressure, is developed as a promising fabrication technique for fused silica due to its deterministic high material removal rate, controllable removal imprint and no mechanical load. The stable and controllable Gaussian-shape removal function makes computer-controlled plasma tool potential to generate complex structures with high accuracy, efficiency and flexibility. In the paper, computer-controlled atmospheric pressure plasma structuring (APPS) is proposed to fabricate 2D/3D patterns on fused silica optics. The capacitively coupled APPS system with a double-layer plasma torch and its discharge characteristics are firstly developed. By means of multi-physics simulation and process investigation, the stable and controllable Gaussian-shape removal function can be achieved. Two different structuring modes, including discrete and continuous APPS, are explored for 2D/3D patterns. A series of structuring experiments show that different kinds of 2D patterns (including square lens array, hexagon lens array and groove array) as well as complex 3D phase plate patterns have been successfully fabricated, which validates the effectiveness of the proposed APPS of 2D/3D patterns on fused silica optics.

## Introduction

Fuse silica with structured and continuous patterns is increasingly applied in high end imaging and illumination fields, due to its abilities to produce particular functional performance^1–5^. These structures can be usually classified into two dimensional (2D) and three dimensional (3D) patterns. Typical 2D patterns include channel array, lens array and pillar array, where the single feature is repeated along lateral directions. For example, lens arrays are widely adopted in light source devices and optical interconnects^6^. 3D patterns have more complex features such as continuous diffractive elements, where the features vary along both lateral and vertical directions. A representative 3D structured optic is continuous phase plate (CPP), which modulates the incident laser to realize beam shaping and thus achieving the uniform illumination of target surface^7^.

Currently, 2D/3D structured optical surfaces are manufactured by different methods such as laser machining^8,9^, micro-milling^10^, diamond turning^11,12^, water jet machining^13^, etc. However, for mechanical machining techniques, the tool wear and failure issues make them difficult to fabricate preferable optical materials such as fused silica^14^. Laser assisted fabrication causes heat-affected zone, which deteriorates the optic surface integrity^15^. Besides, most technologies above are costly in terms of equipment and of low removal efficiency for most optical materials^8,16^. Fused silica structured optics are highly demanded due to its excellent optical, chemical and mechanical properties. Fluorine-based atmospheric pressure plasma processing (APPP) has been reported as a cost-effective technique to fabricate fused silica optics because of its deterministic high material removal rate. Also, it is based on pure chemical reaction between silicon-based materials surface atom and reactive fluorine radicals generated by the plasma at atmospheric pressure, which avoids any introduction of damage to the processed surface and significantly lowers the processing cost as well. Jourdain et al.^17^ adopted the reactive atom plasma process for figuring of large fused silica optics. An inductively coupled plasma (ICP) torch with De-Laval nozzle was applied to generate a Gaussian removal footprint. To reduce the thermal effect, an adapted tool-path strategy was combined with an iterative figuring procedure. More commonly, capacitively coupled plasma is used in APPP. Takino et al.^18^ investigated chemical vaporization machining (CVM) with radio frequency plasma with a pipe electrode in an atmospheric environment. The removal rate of plasma CVM was 4 to 1100 times faster than that of polishing, while the roughness of the processed surfaces was almost the same as that of the polished surfaces. Li et al.^19^ modelled the APPP discharge process and surface chemical reaction using the multi-physics simulation. Gaussian removal profile was mainly determined by the distribution of active F atoms, and the ratio of O/CF_x_ was the key factor affecting the surface morphology formation. Comprehensive characterization of surface topography indicated the existence of cellular microstructures after APPP, which caused opacification phenomenon^20^. Arnold et al.^21^ proposed the atmospheric plasma jet machining based manufacturing chain (comprising plasma polishing, plasma jet based figuring and correction, and soft polishing) for freeform silica optical elements. In addition, a finite element (FE) heat transfer model was used to compensate spatio-temporal variations of surface temperature and nonlinear material removal. The form error convergence was improved by an iterative correction of the targeted removal according to FE modelling.

Most of the aforementioned studies mainly aim for material investigation and form error correction using APPP techniques. The tool influence function was obtained experimentally and iterative figuring was needed to converge the surface error to specification. However, little detailed research has been carried out on fabrication of different patterned surfaces using APPP. The stable and controllable Gaussian-shape removal function makes computer-controlled APPP potential to fabricate 2D/3D patterns with high accuracy, efficiency and flexibility.

This paper presents the development of computer-controlled atmospheric pressure plasma structuring (APPS) method for 2D/3D patterns on fused silica optics. Firstly, a capacitively coupled APPS system is introduced. By means of finite element simulation and process investigation, the discharge, flow field and removal characteristics are then presented. Two different structuring modes, including discrete and continuous APPS, are respectively explored for 2D/3D patterns generation. Surface generation principle and simulation of different structuring modes are presented in detail. Finally, a series of experiments are carried out to validate the effectiveness of the proposed computer-controlled APPS for 2D/3D patterns on fused silica optics.

## APPS system, characteristics and process investigation

### APPS system and plasma torch

The overall APPS system consists of a plasma torch, a gas supply module and motion workbench, which has been described in our previous work^22^. The plasma (He and O_2_) is generated by radio frequency RF power which forms a chemical reactor; the reactant gas (CF_4_) fed into the reactor is decomposed to reactive radicals (F) by the collision with plasma electrons. These reactive radicals diffuse to the fused silica surface, and the material removal is accomplished as the reaction product is volatile. The balanced chemical reaction equation is ${\text{SiO}}_{2} + {\text{CF}}_{4} \to {\text{SiF}}_{4} \uparrow + {\text{CO}}_{2} \uparrow$.

**Figure 1:**
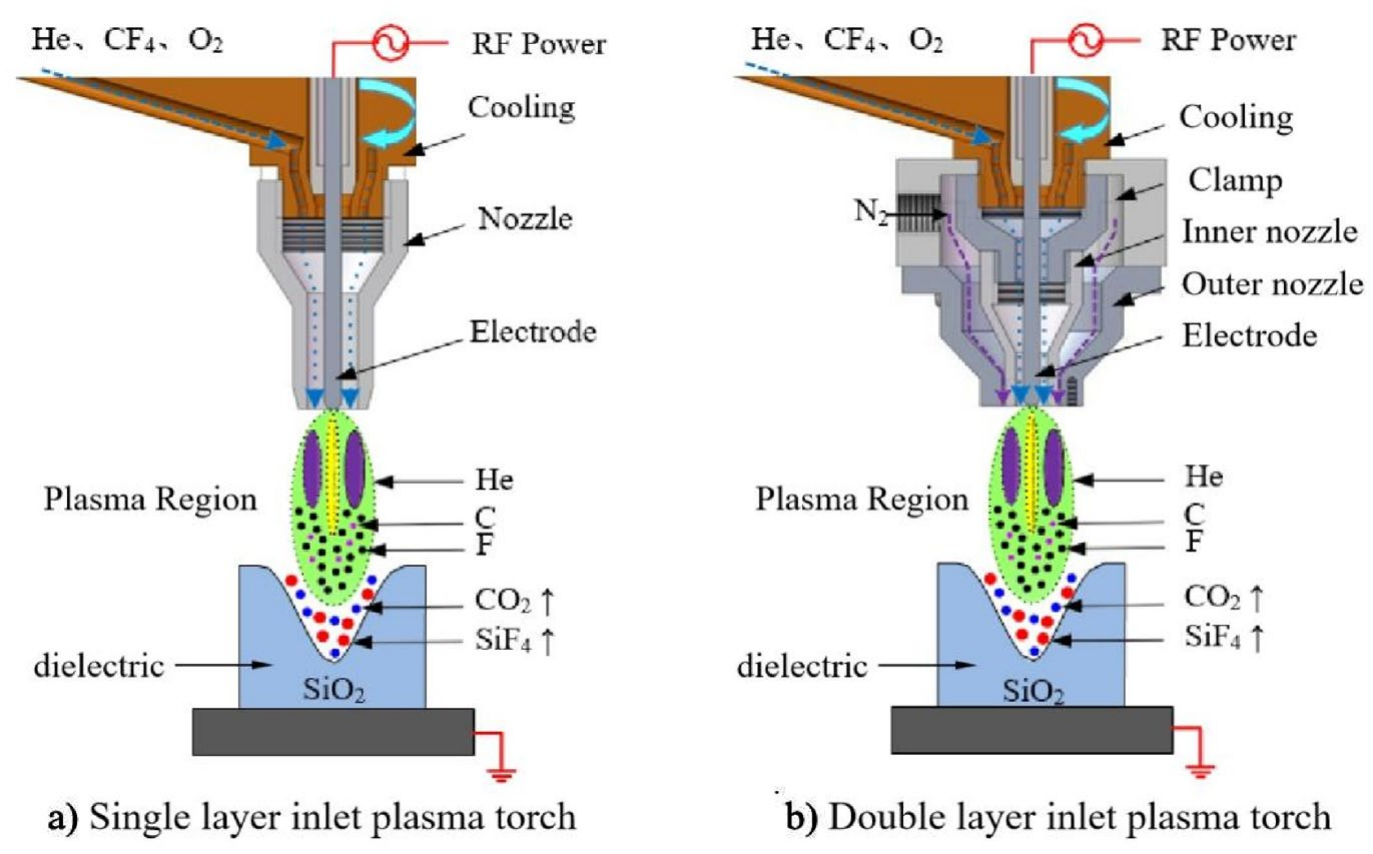
Schematic diagram of a single-layer and double-layer plasma torch.

Based on our experiments, it is found that the plasma flame tends to be interfered by the external environment such as the exhaust system and surrounding air environment, which would affect the stability of the plasma. In this work, a double-layer plasma torch is designed to shield the external airflow from the plasma. A schematic diagram of a single-layer and double-layer plasma torch is shown in Fig. 1.The new double-layer plasma torch consists of the base, clamp, ceramic inner nozzle, ceramic outer nozzle, and aluminum electrode. The plasma torch adopts a double-layer coaxial structure. The inner layer is connected with the plasma gas He, the reaction gas CF_4_ and the catalytic gas O_2_. And the outer layer is provided with the shielding gas N_2_ to protect the plasma flame during the processing. The plasma torch is effectively cooled by circulating water and air.

### Discharge characteristics

The plasma torch designed in this work is based on dielectric barrier discharge (DBD) principle. In the field of atmospheric plasma processing, it is desirable to obtain a dielectric barrier uniform discharge, which is essentially a glow discharge rather than filament discharge. After ignition, atmospheric pressure glow discharge presents in two modes: alpha α mode and beta β mode. In the alpha α mode, electrons are excited throughout the discharge region; in the beta β mode, most of the input power is consumed in the plasma sheath near the electrodes and is not used to excite the plasma. For the two modes, only the alpha α mode can produce a stable and uniform plasma, while the beta β mode is easily converted into an unstable filament discharge which is not suitable for APPS.

In order to experimentally analyze the discharge characteristics of the plasma torch, a current probe (Tektronix P6021) and a high voltage probe (Tektronix P6015A 1000X) are adopted to simultaneously detect the current and voltage of the plasma torch discharge. The sampled data is collected using an oscilloscope (Agilent InfiniiVision DSO-X 2022A Technologies) with sampling frequency 2GSa/s and bandwidth 100 MHz.

**Figure 2:**
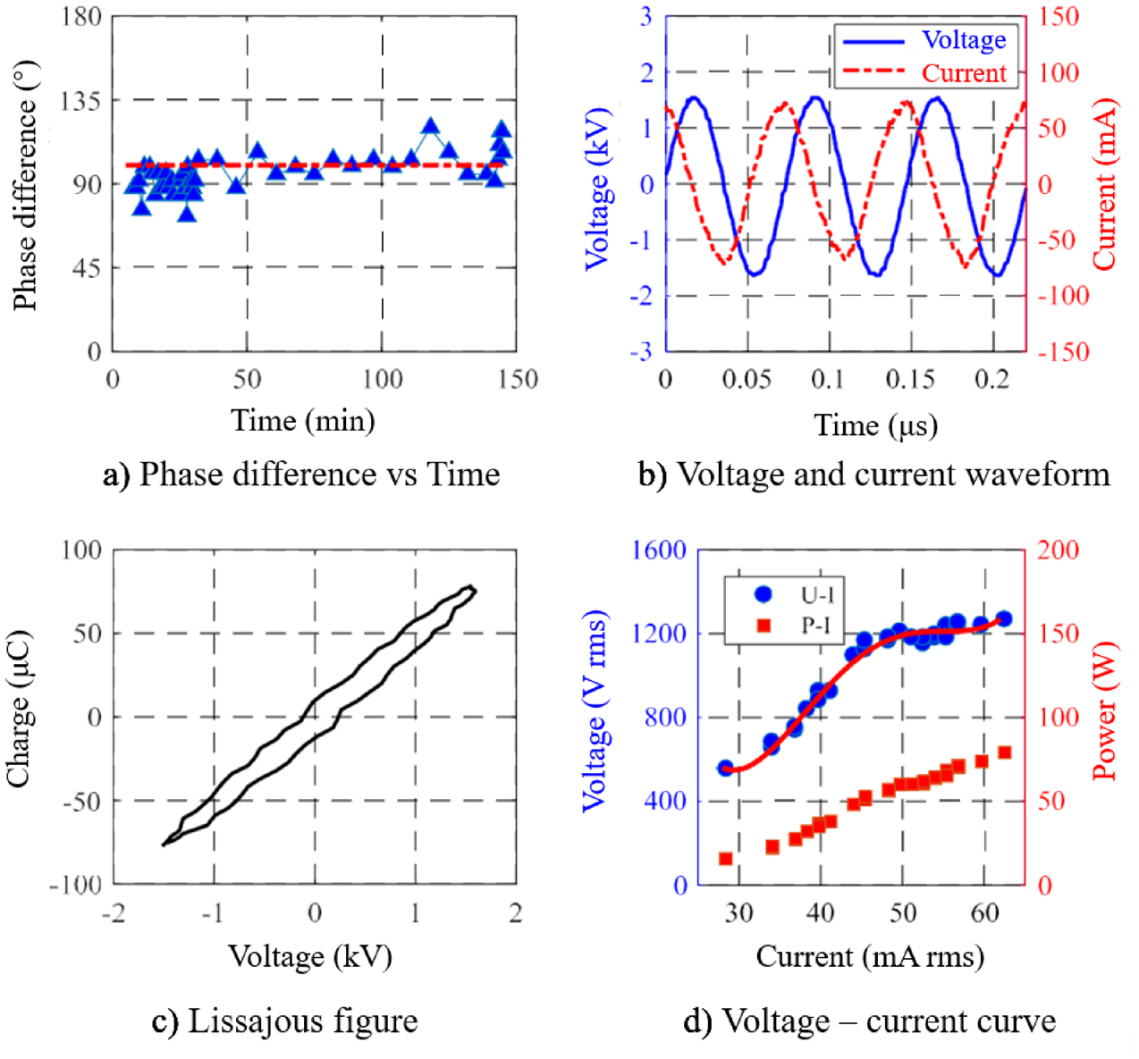
Discharge characteristics of the APPS torch.

The experimental results are shown in Fig. 2. Figure 2a,b show that the phase of the current waveform leads the phase of voltage waveform about 100° and does not change much with time, indicating that the electric load is capacitive and stable. It can be seen from Fig. 2c that the calculated Lissajous figure has a smooth transition at both ends, and there are multiple curve steps on the left and right sides, indicating that the discharge of the plasma torch belongs to atmospheric pressure glow discharge. As illustrated in Fig. 2d, at the beginning of ignition, as the input power increases, the voltage and current increase linearly until the plasma begins to discharge (when the power is around 50 W). At this time, the input power continues to increase, and the voltage remains basically unchanged with the increase of the current, showing that the discharge mode of the plasma torch belongs to the alpha α mode.

### Simulation of flow field and molar ratio spatial distributions

**Figure 3:**
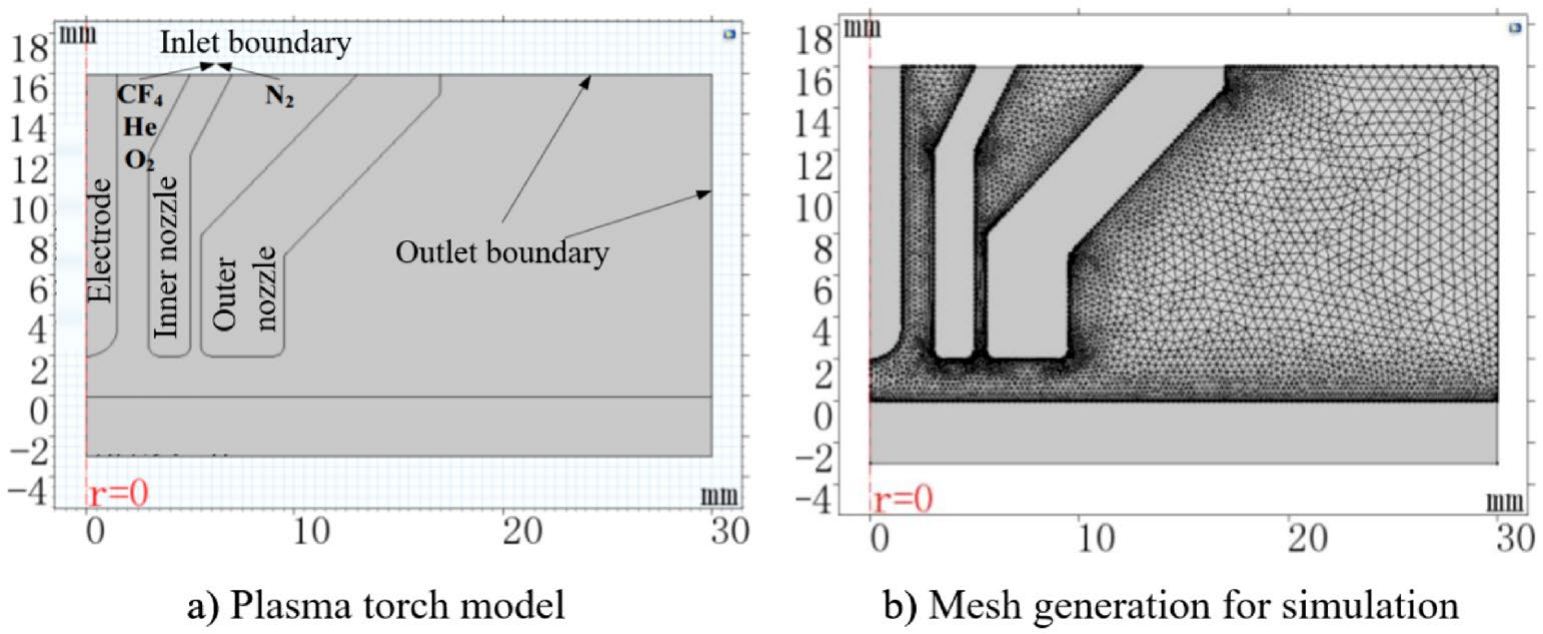
Simulation model of APPS plasma torch and its mesh generation.

In order to explore the gases distribution during APPS and verify the design of the new plasma torch, the flow filed and species distribution is simulated using finite element analysis in COMSOL Multiphysics software. The simulation model of the APPS plasma torch is illustrated in Fig. 3. In order to simplify the calculation process, a two-dimensional axisymmetric model is established. Since the flow direction of the gas changes sharply at the tip of the electrode, denser meshes are generated in this region, as shown in Fig. 3b.

Boundary conditions are configured as follows. For flow field simulation, the inlet boundary condition is set as the mass flow inlet and the outlet boundary condition is set as the pressure outlet (relative pressure is zero). Other boundaries are set as wall by default. When fluid flow is coupled with mass transfer, it is important to suppress reflow, which ensures mass conservation as well as fast and robust convergence. As for the solver configuration, the flow field simulation model selects a steady-state incompressible single-phase flow model. The flow field distribution of the gas is obtained by solving the Navier–Stokes equation. The input parameters in the simulation are listed in Table 1.

**Table 1:** Input parameters in the simulation.

Input parameters	He	CF_4_	O_2_	N_2_
Flow rate	670 (sccm)	113.6 (sccm)	20 (sccm)	1000 (sccm)
Molar mass *M*_*i*_ (1/mol)	4.003	88.01	32	28.02
Dynamic viscosity *μ*_*i*_ (× 10^−5^ Pa s)	1.89	1.763	2.019	1.74

**Figure 4:**
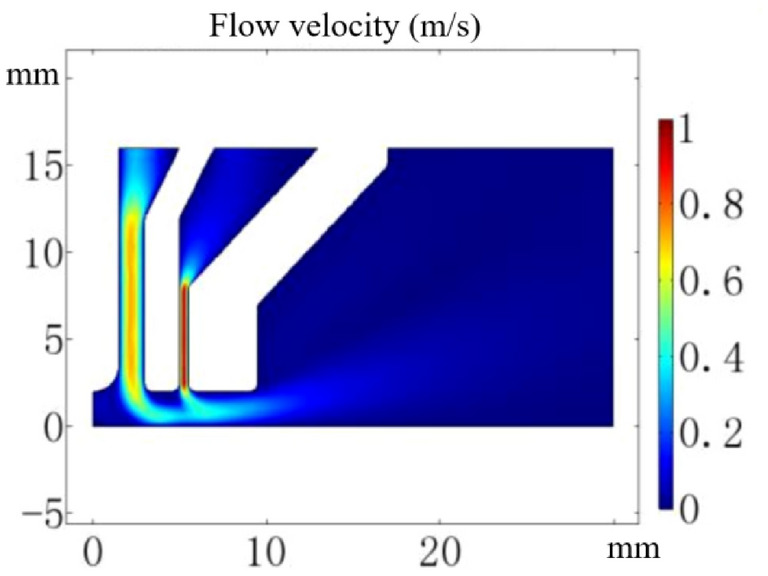
Flow filed simulation result.

The flow field distribution of the gas is obtained and the result is shown in Fig. 4. The maximum gas flow rate is 1 m/s. The Reynolds number of the gas is about 169, which means that it is reasonable to use the laminar flow model in the single-phase flow. After the gas flows out of the nozzle, the velocity direction changes to the horizontal direction that is the radial direction of the plasma torch. Out of the nozzle range, the flow field begins to diverge and the flow rate decreases.

**Figure 5:**
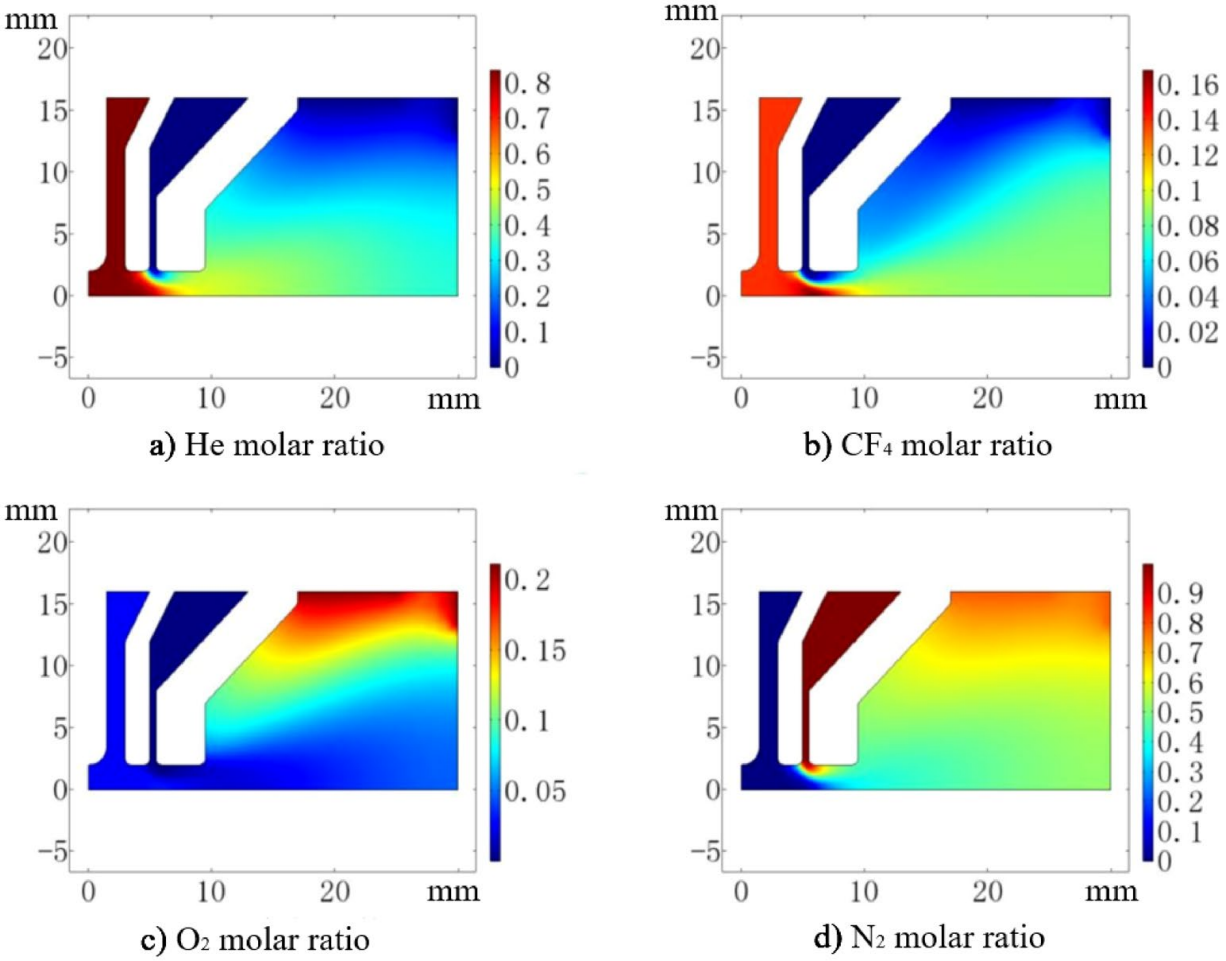
Molar ratio spatial distributions of He, CF_4_, O_2_ and N_2_.

To simulate the spatial distribution of the various gas components, the Transport of Concentrated Species (TCS) model in COMSOL Multiphysics software is used. The inlet boundary condition of the plasma torch is set as Mole fractions-inflow, and the outlet boundary condition is set as Open boundary. The spatial distribution of the various particles (He, CF_4_, O_2_ and N_2_) is obtained by solving the Maxwell–Stefan equation. The calculated molar ratio distribution of He, CF_4_, O_2_ and N_2_ gas components is shown in Fig. 5. It can be seen that the gas molecules are uniformly distributed in the plasma discharge region, which is the same as the mole fraction of the gas at the inlet. In addition, the outer protective gas N_2_ does not affect the active particle distribution in the plasma region.

### Process investigation on removal function

**Figure 6:**
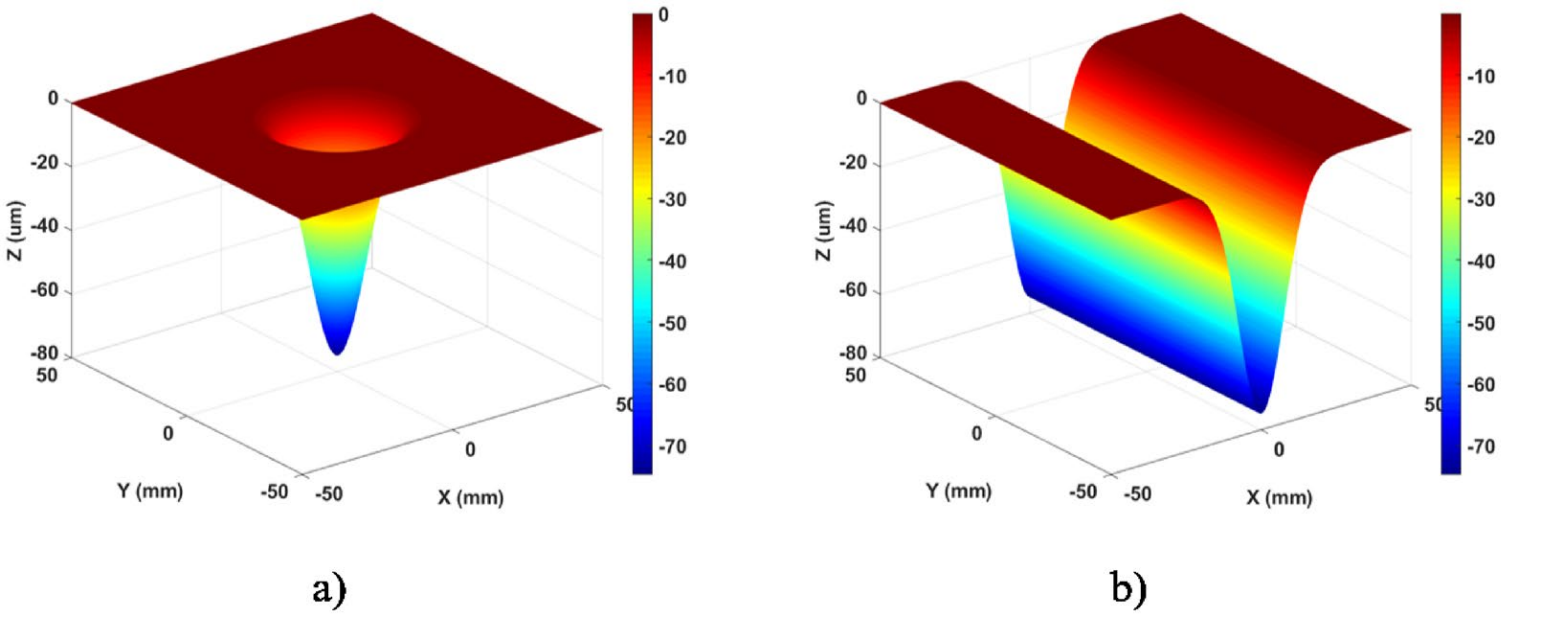
Schematic of (**a**) static removal function and (**b**) scanning removal function (groove).

Atmospheric processing plasma is a complex physical and chemical process, and its material removal rate is affected by many factors, such as plasma gas flow, reactant gas flow, RF power applied, etc. In this section, the single-factor method is used to analyze the effects of process parameters on removal characteristics of the designed double nozzle plasma torch. Preliminary process investigation is based on groove machining (scanning removal function) by atmospheric plasma with different processing parameters. The schematic of static removal function and scanning removal function (for groove) of APPS is shown in Fig. 6.

**Figure 7:**
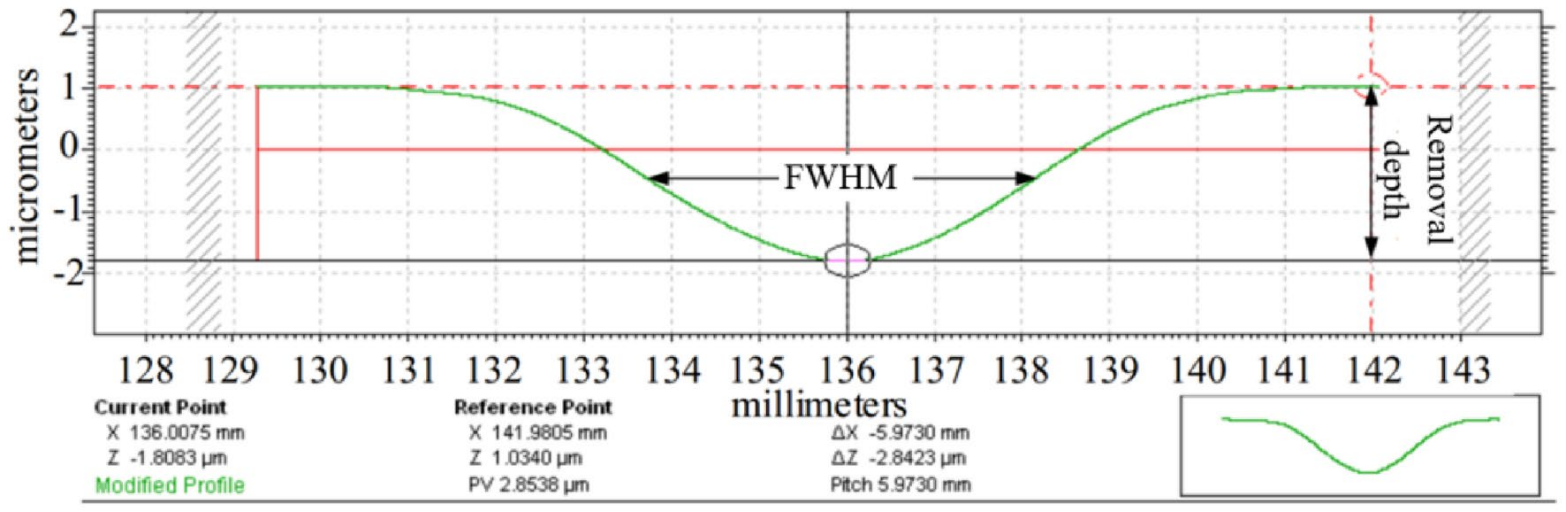
Typical removal profile by APPS.

Unless otherwise specified, the groove scanning speed is 1 mm/s, and the sample used in the experiment is 100 × 100 × 3 mm fused silica and the shielding gas N_2_ flow rate is set as 1000 sccm. The machined groove geometry (cross-section profile) is measured by a stylus profilometer (Taylor Hobson Form Talysurf PGI 1240). Figure 7 shows the typical removal function profile, which is mathematically fitted by Gaussian function in Eq. (1).

In APPS, the removal rate and the full width at half maximum (FWHM) are generally used to describe the processing capability of the removal function. The removal rate is defined as the depth of removal per unit time (defined as Eq. (2)), and the FWHM is defined as the width in the horizontal direction at half of the removal depth *R*/2 (defined as Eq. (3)). The volume removal rate is used to estimate the efficiency of plasma processing (defined as Eq. (4)).

1 $$r\left( {x,y} \right) = R \cdot e^{{ - \frac{{x^{2} + y^{2} }}{{2\sigma^{2} }}}}$$

2 $$a = \frac{R}{t}$$

3 $${\text{FWHM}} = 2\sqrt {2\ln 2} \sigma$$

4 $$V_{k} = \iint {ae^{{ - \frac{{x^{2} + y^{2} }}{{2\sigma^{2} }}}} dxdy = \int\limits_{0}^{2\pi } {\int\limits_{0}^{ + \infty } {ae^{{ - \frac{{r^{2} }}{{2\sigma^{2} }}}} rd\theta } } dr = 2\pi a\sigma^{2} } = \frac{\pi }{4\ln 2}aFWHM^{2}$$

where *R* is removal depth, *a* is removal rate and *σ* is standard deviation of Gaussian function.

#### The effect of processing time on removal function

The relationship between the material removal rate and the processing time directly affects the complexity and accuracy of the APPS algorithm. Linear relationship between the material removal and the processing time is highly preferred. Therefore, the processing time is used as a variable to investigate the variation of the removal rate with time.

**Figure 8:**
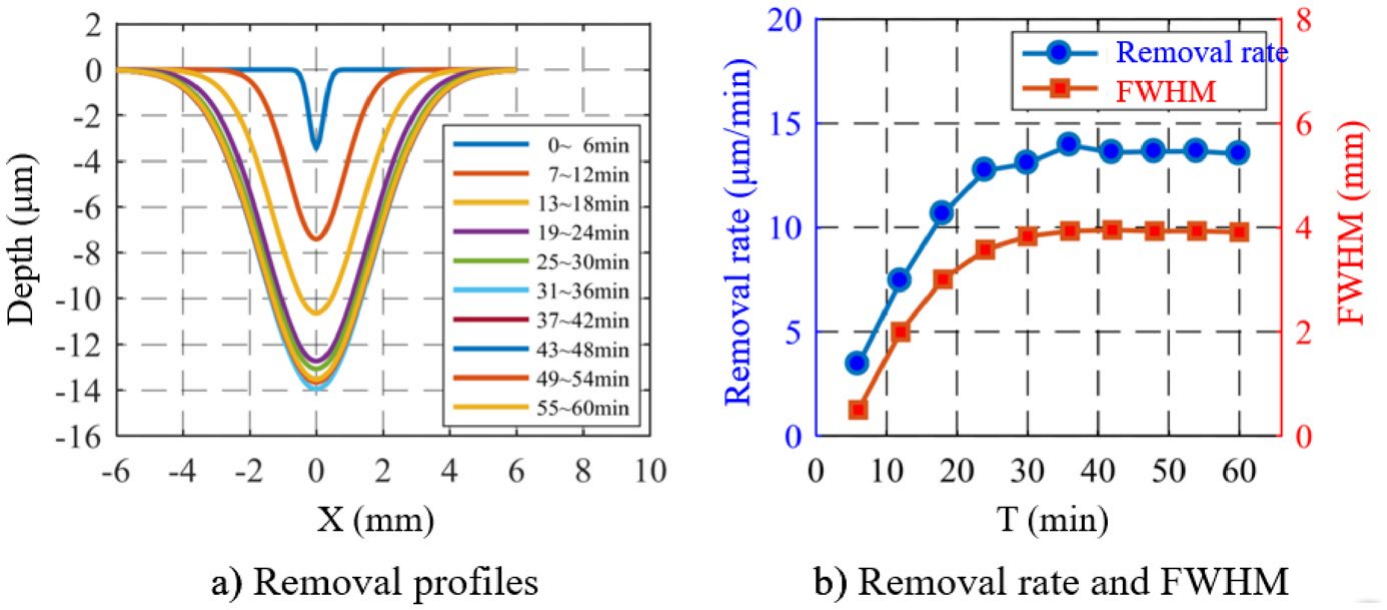
Effect of processing time on removal rate and FWHM.

In this experiment, 10 grooves are fabricated on a fused silica substrate. For each groove, the scanning speed is 1 mm/s and the scanning length is 60 mm (reciprocating three times). Thus, the total processing time is 60 min. The other processing parameters are shown in Table 2, and the experimental results are shown in Fig. 8. It can be seen that within 0–30 min, the removal rate and the FWHM increase with time. After 30 min, the removal function tends to be stable, and the removal rate and the FWHM are constant. The nonlinear phenomenon is attributed to the warm up of the power system and the fact that electrode and processed surface region does not reach thermal equilibrium. At the beginning of the processing, the temperature of the electrode surface is relatively low. As the time goes on, it gradually increases and then reaches the equilibrium state. Therefore, the discharge gradually becomes stable, leading to the saturation of the removal rate.Therefore, it is necessary to stabilize the plasma torch for more than 30 min before the actual processing.

**Table 2:** Processing time as processing variable.

He flow (sccm)	CF_4_ flow (sccm)	O_2_ flow (sccm)	Distance (mm)	Power (W)
670	113.6	20	2	100

#### The effect of He flow rate on removal function

The main role of the inert gas He is to produce a stable plasma discharge that provides an environment for the excitation of active F atoms. The energy of helium atomic He(23S) is 19.8 eV, which is higher than the first ionization energy of most gas molecules. Uniform dielectric barrier discharge of He can be easily generated in atmospheric pressure. In addition, the He flow accounts for the majority of the inner mixed gas, and is an important factor in determining the velocity distribution, the concentration and distribution of the active F atom. The processing parameters listed in Table 3 are used to analyze the effect of He flow rate on the removal function.

**Table 3:** He flow rate as processing variable.

He flow (sccm)	CF_4_ flow (sccm)	O_2_ flow (sccm)	Distance (mm)	Power (W)
340, 670, 1010, 1350, 1680, 2020	113.6	20	2	100

**Figure 9:**
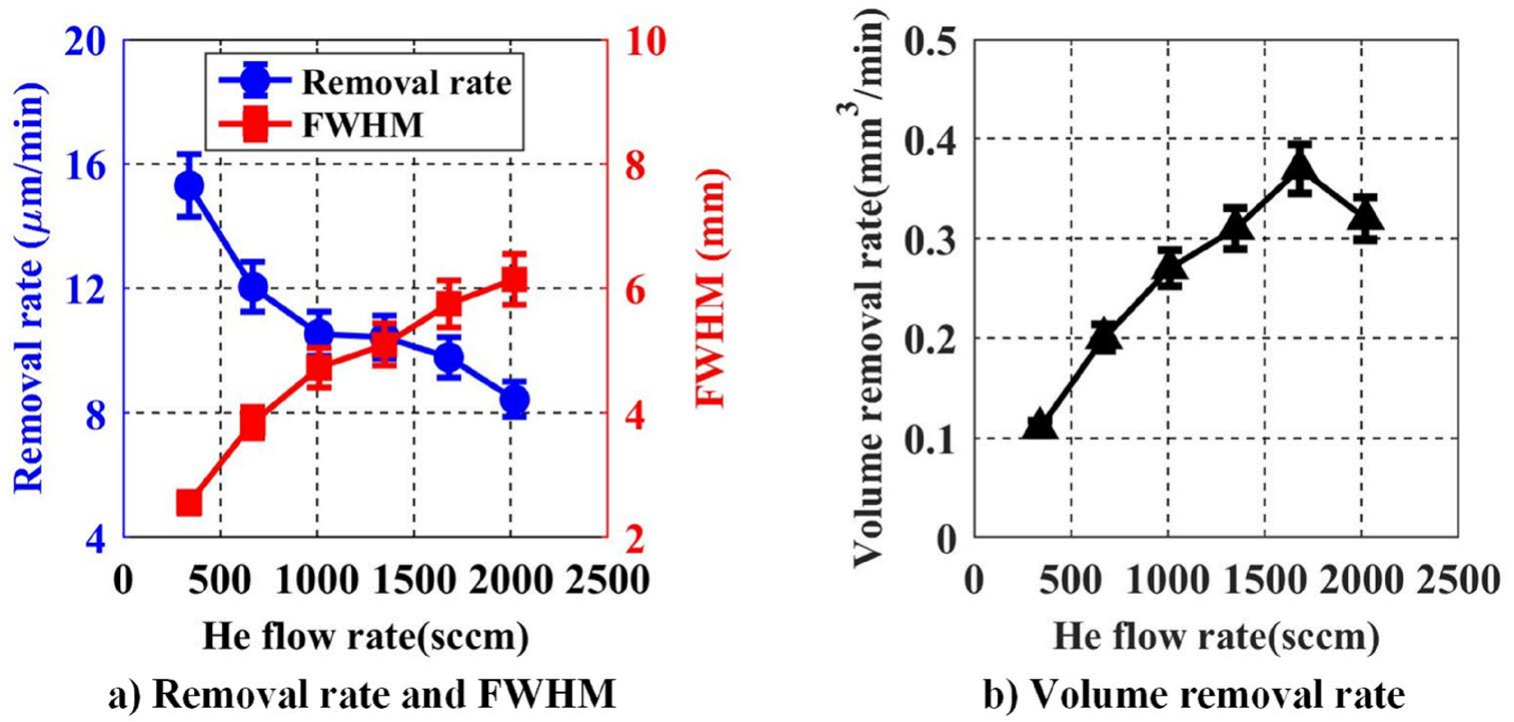
Effect of He flow rate on removal rate and FWHM.

The experimental results are shown in Fig. 9. As the He flow rate increases, the FWHM increases, while the peak removal rate decreases. In Fig. 9b, as the He flow increases, the volume removal rate first increases and then decreases. When the He flow rate is 1700 sccm, the maximum value reaches 0.37 mm^3^/min. Based on the chemical reaction characteristics and the Le Châtelier's principle, with the increase of He flow rate, the concentration of active F atoms gradually decreases, so the peak removal rate decreases. However, the distribution range and the total amount of active F atoms gradually increase. As a result, the FWHM and volume removal rate will increase. When the He flow increases to a certain range, the number of active F atoms reaches saturation, but the concentration still decreases, and the volume removal rate begins to drop. Therefore, in the actual processing, He flow rate is selected according to the processing efficiency and resolution requirements.

#### The effect of CF4 flow rate on removal function

The reaction gas in the APPS system is CF_4_, and its main role is to generate a large amount of active F atoms through electron collision reaction and to react with the fused silica, thereby achieving the material removal. The processing parameters listed in Table 4 are used to investigate the effect of CF_4_ flow rate on the removal function.

**Table 4:** CF_4_ flow rate as processing variable.

He flow (sccm)	CF_4_ flow (sccm)	O_2_ flow (sccm)	Distance (mm)	Power (W)
670	63.64, 79.55, 95.45, 103.41, 111.36, 127.27	20	2	100

**Figure 10:**
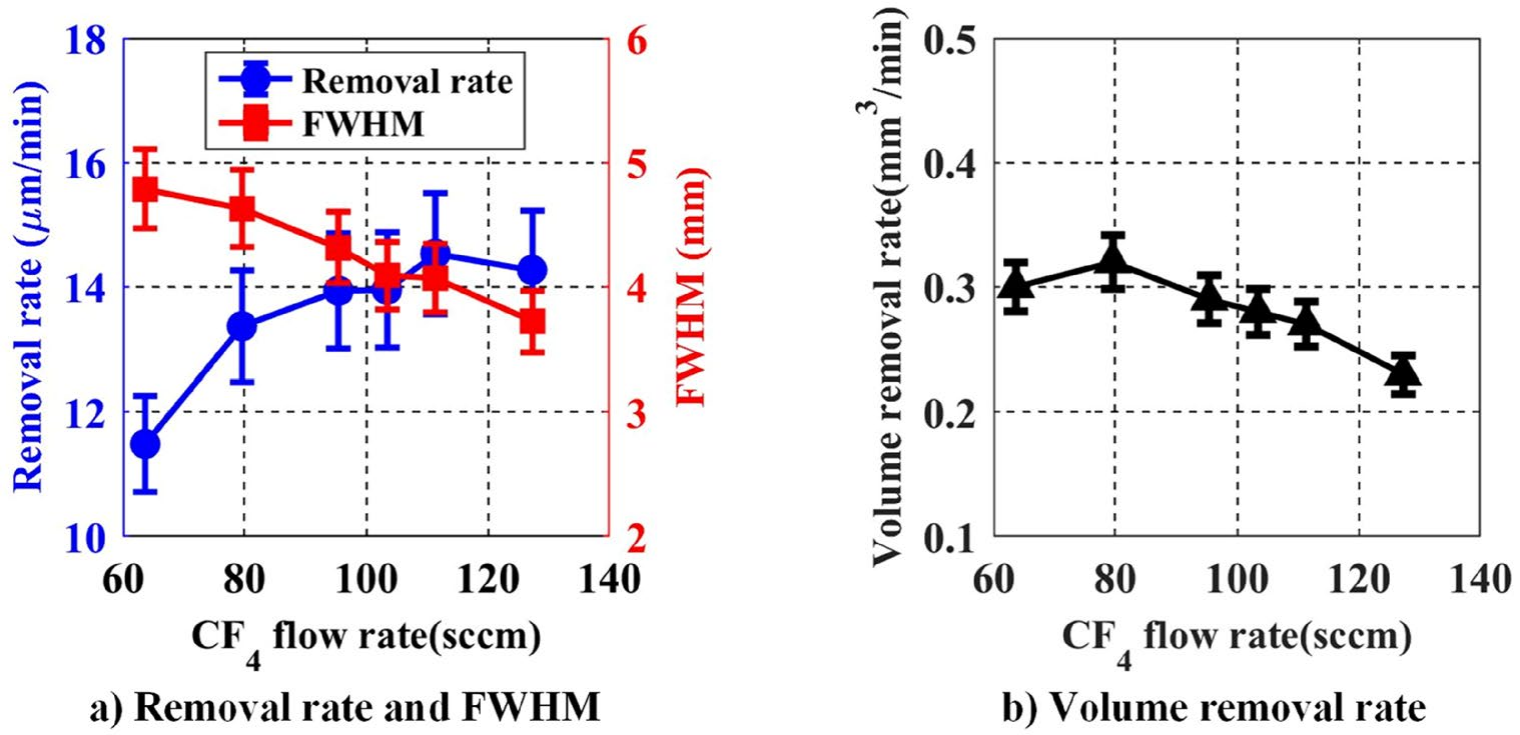
Effect of CF_4_ flow rate on removal rate and FWHM.

The experimental results are shown in Fig. 10. As the flow rate of CF_4_ increases, the concentration of active F atoms gradually increases, and thus the removal rate increases. However, the FWHM and volume removal rate of the removal function gradually reduces. This may be due to the fact that CF_4_ itself is an insulating gas and does not generate discharge. Therefore, as the flow rate of CF_4_ further increases, the plasma discharge region gradually shrinks, and thus FWHM of the removal function decreases. It can be seen from the Eq. (4) that the effect of the FWHM contributes more to the volume removal rate than the removal rate. As a result, the volume removal rate reduces.

#### The effect of RF power on removal function

The RF power determines the amount of energy input into the plasma system and is an important parameter affecting the plasma excitation intensity as the plasma discharge is driven by RF electric field. When the input RF power applies, electric energy is transferred to the electrons, and the heavy particles exchange energy efficiently only by colliding with the neutral gas molecules. The processing parameters listed in Table 5 are used to investigate the effect of RF power on the removal function.

**Table 5:** RF power as processing variable.

He flow (sccm)	CF_4_ flow (sccm)	O_2_ flow (sccm)	Distance (mm)	Power (W)
670	111.36	20	2	70, 80, 90, 100, 110, 120

**Figure 11:**
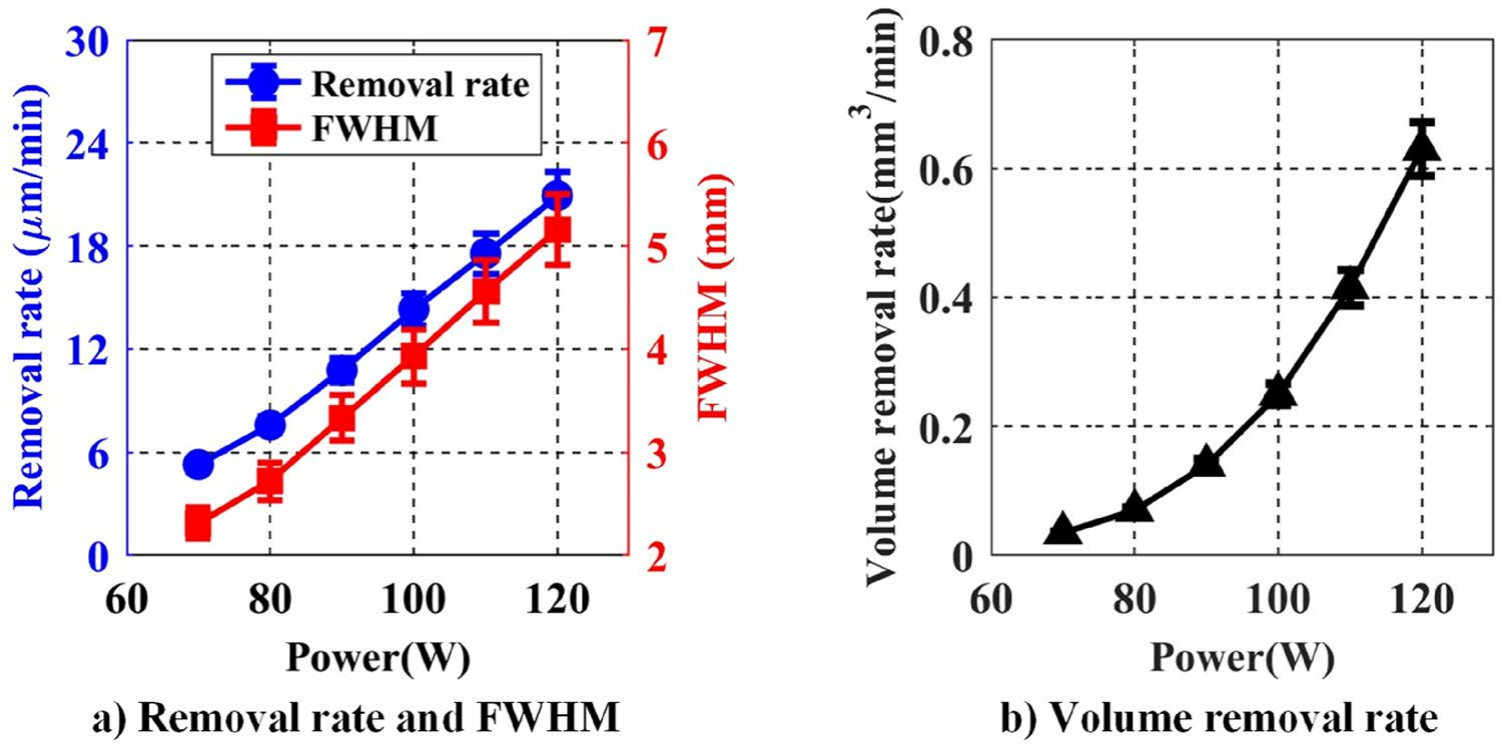
Effect of RF power on removal rate and FWHM.

The experimental results are shown in Fig. 11. It can be seen that the removal rate and the FWHM both increase approximately linearly as the input power increases. This is because more electrons are excited and accelerated, causing a more intense collision reaction with heavy particles, which in turn produces more active F atoms. The concentration and distribution range are both increased, so the removal rate and the FWHM increase accordingly. However, when the input RF power is too high, the He glow discharge at atmospheric pressure, tends to convert into arc discharge, which would damage the electrode and be unacceptable for APPS. Therefore, the input RF power needs to be carefully controlled within an appropriate range.

## Discrete and continuous APPS modes

With the controllable Gaussian-shape removal function, APPP is able to structure 2D/3D patterns with high accuracy, efficiency and flexibility. Two different structuring modes, including discrete and continuous APPS, are respectively proposed for 2D/3D pattern generation. Surface generation principle and experiments of the two structuring modes are also presented in the following section.

### Discrete APPS of 2D patterns

**Figure 12:**
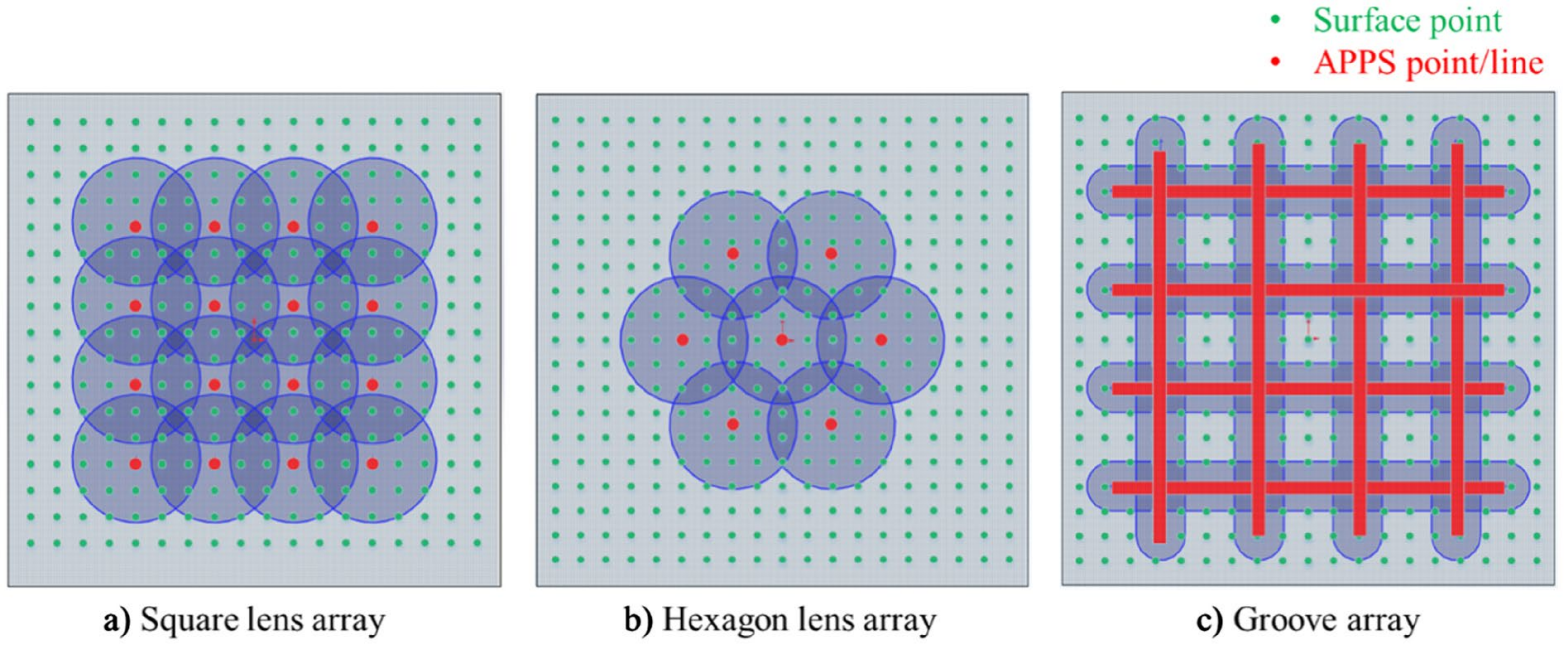
Schematic of discrete APPS of different 2D patterns.

Typical 2D patterns include groove array, lens array and pillar array, etc., where the single feature is repeatedly distributed along lateral directions in 2D plane. In this work, the controllable APPP removal function is used to generate a single feature on the fused silica substrate. By means of controlling the dwell position grid or trajectory, the single feature, such as spot and groove, can be replicated to generate 2D patterns. In this mode, APPS grid is not relevant to the grid of surface points. Normally, the distribution of dwell position/scanning line is discrete on the processed surface. 2D structured surface is thus generated by the imprint of APPP removal function according to the design pattern. The features can be overlapped or non-overlapped with each other. The schematic of APPS of different 2D patterns is illustrated in Fig. 12.

**Figure 13:**
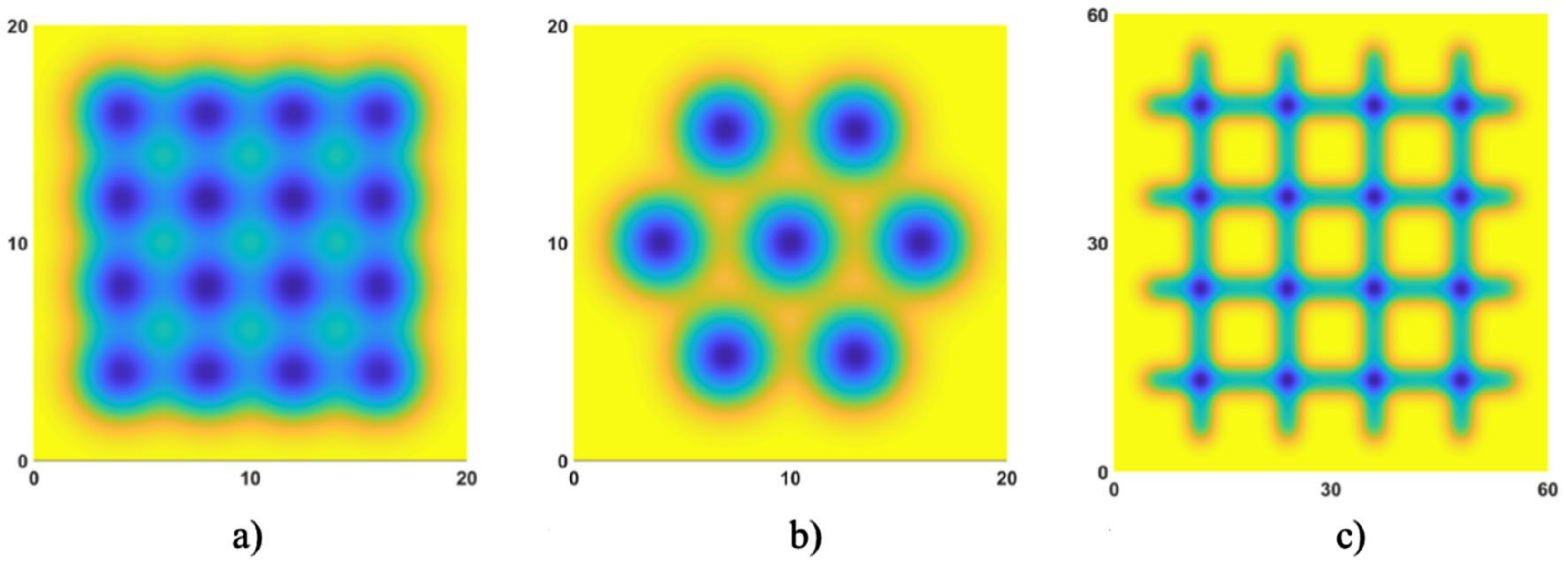
Simulation of discrete APPS of 2D patterns (**a**) Square lens array (**b**) Hexagon lens array (**c**) Groove array.

Simulation of discrete APPS is also carried out to predict the generation of 2D patterned surface, including square lens array, hexagon lens array, and groove array. For the removal function used in the simulation, the removal rate is 19.69 μm/min, and the FWHM is 3.28 mm. For the 4 × 4 square lens array, the spacing is set as 4 mm; for the hexagon distributed lens array, the spacing is set as 6 mm; for the groove array, the spacing between each groove is set as 12 mm, and the scanning velocity is set as 1 mm/min. The surface generation results of different patterns are illustrated in Fig. 13.

**Figure 14:**
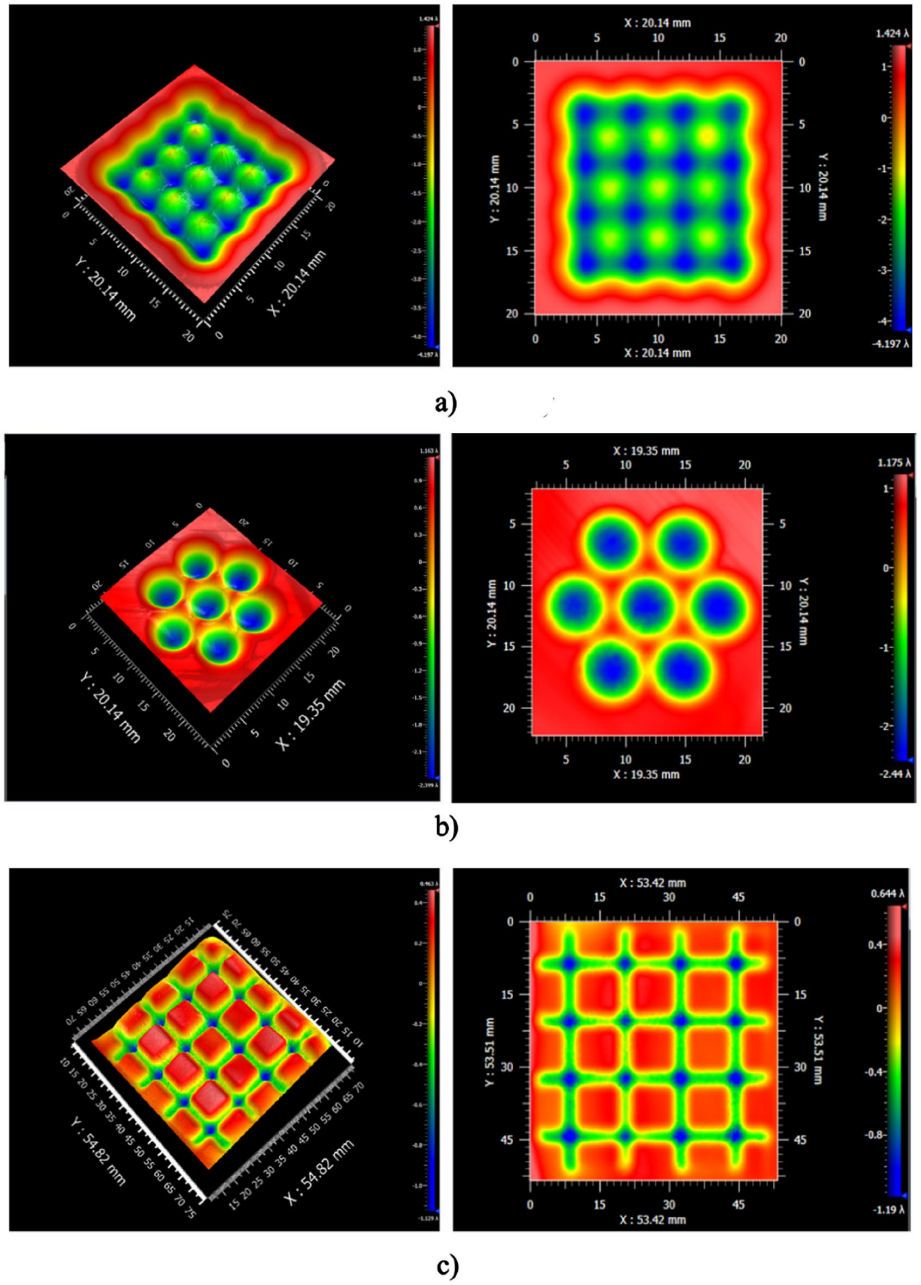
Discrete APPS experiment results: (**a**) Square lens array (**b**) Hexagon lens array (**c**) Groove array.

To verify the discrete APPS mode, the experimental structuring of 2D patterns (including lens array and groove array) was performed according to the discussion above. The fabricated 2D patterns on the fused silica substrate was measured by a Zygo phase shifting interferometer and the results are shown in Fig. 14. From experimental results, different kinds of 2D patterns, including square lens array, hexagon lens array and groove array, were successfully fabricated on fused silica by means of discrete APPS and agreed well with the simulation results.

### Continuous APPS of 3D patterns

**Figure 15:**
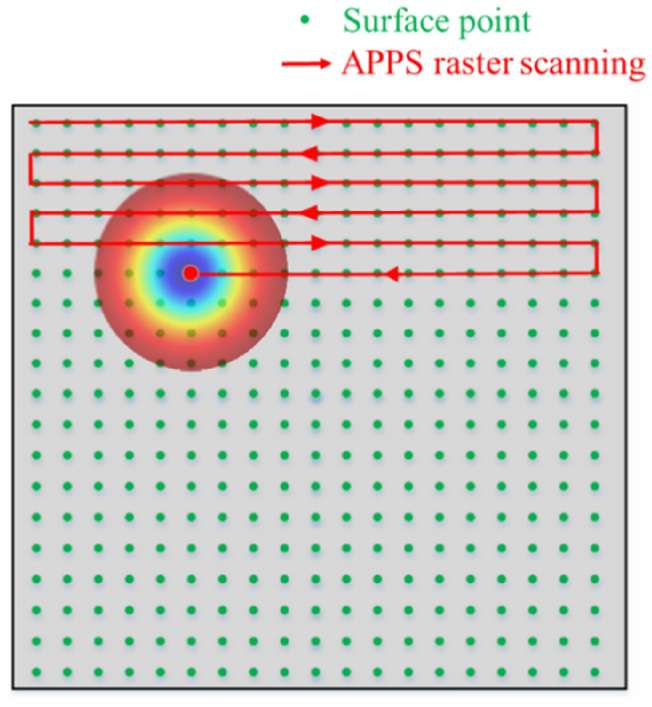
Schematic of continuous APPS mode.

Continuous APPS mode can be used for the generation of 3D patterns, where the features can vary along both lateral and vertical directions in 3D space. Besides the positioning and trajectory control, dwell time of plasma torch is added as another process parameter to control the surface generation. In this mode, APPS dwell time grid is equal to the grid of surface points. The structuring process is considered as continuous in terms of scanning trajectory with varying dwell time. Preferably, the raster scanning is adopted in consideration of motion control accuracy and data processing consistency. A schematic of continuous APPS mode is illustrated in Fig. 15.

Mathematically, the desired removal distribution is the convolution ($\otimes$) between the removal function and control dwell time matrix. It can be expressed by the mathematical model as shown in the following equation,

5 $$TR(x,y) = r(x,y) \otimes dT(x,y)$$

where $TR(x,y)$ is target removal distribution for 3D patterns, $r(x,y)$ is APPS removal function and $dT(x,y)$ is the dwell time matrix. Therefore, for given removal function of plasma torch, arbitrary 3D continuous patterns can be generated on the substrate by means of controlling the APPS dwell time. If the continuous pattern data is known and the removal function is experimentally obtained, the dwell time matrix is solved by linear equation method (also known as deconvolution process) with high accuracy.

**Figure 16:**
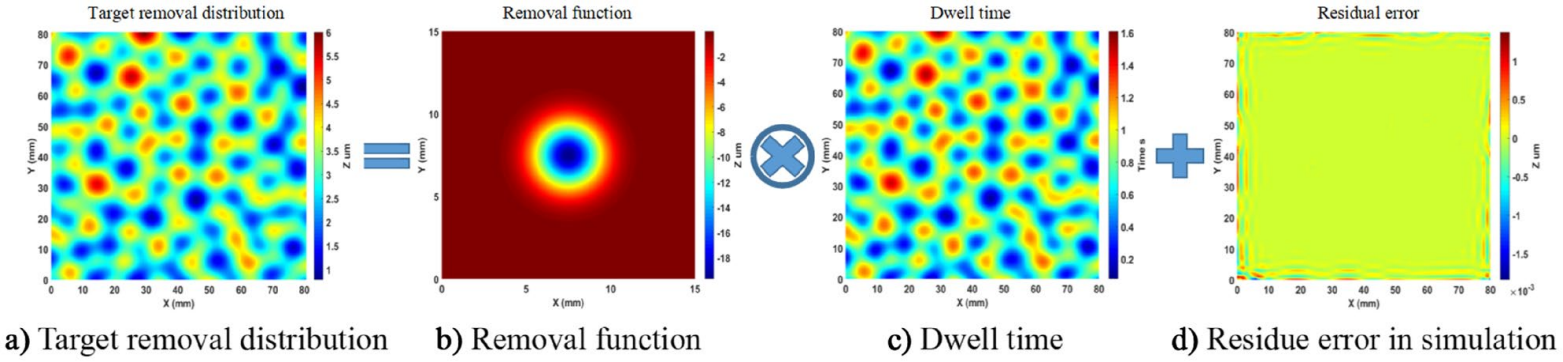
Simulation of continuous APPS of 3D CPP pattern.

Simulation of continuous APPS is performed on a representative 3D structured surface, continuous phase plate (CPP), where the 3D features vary along both lateral and vertical directions. The surface size is 80 mm × 80 mm and the PV value is 5.22 μm. For the removal function used in the simulation, the removal rate is 19.69 μm/min and the FWHM is 3.28 mm. In addition, residue error is calculated as the difference between the design data and simulated removal (convolution of removal function and solved dwell time). The simulation results, including dwell time and residue error, are shown in Fig. 16. The residue error in the simulation is negligible (as the PV and RMS values of the error are 3.2 nm and 0.15 nm, respectively), which verifies the continuous APPS model.

**Figure 17:**
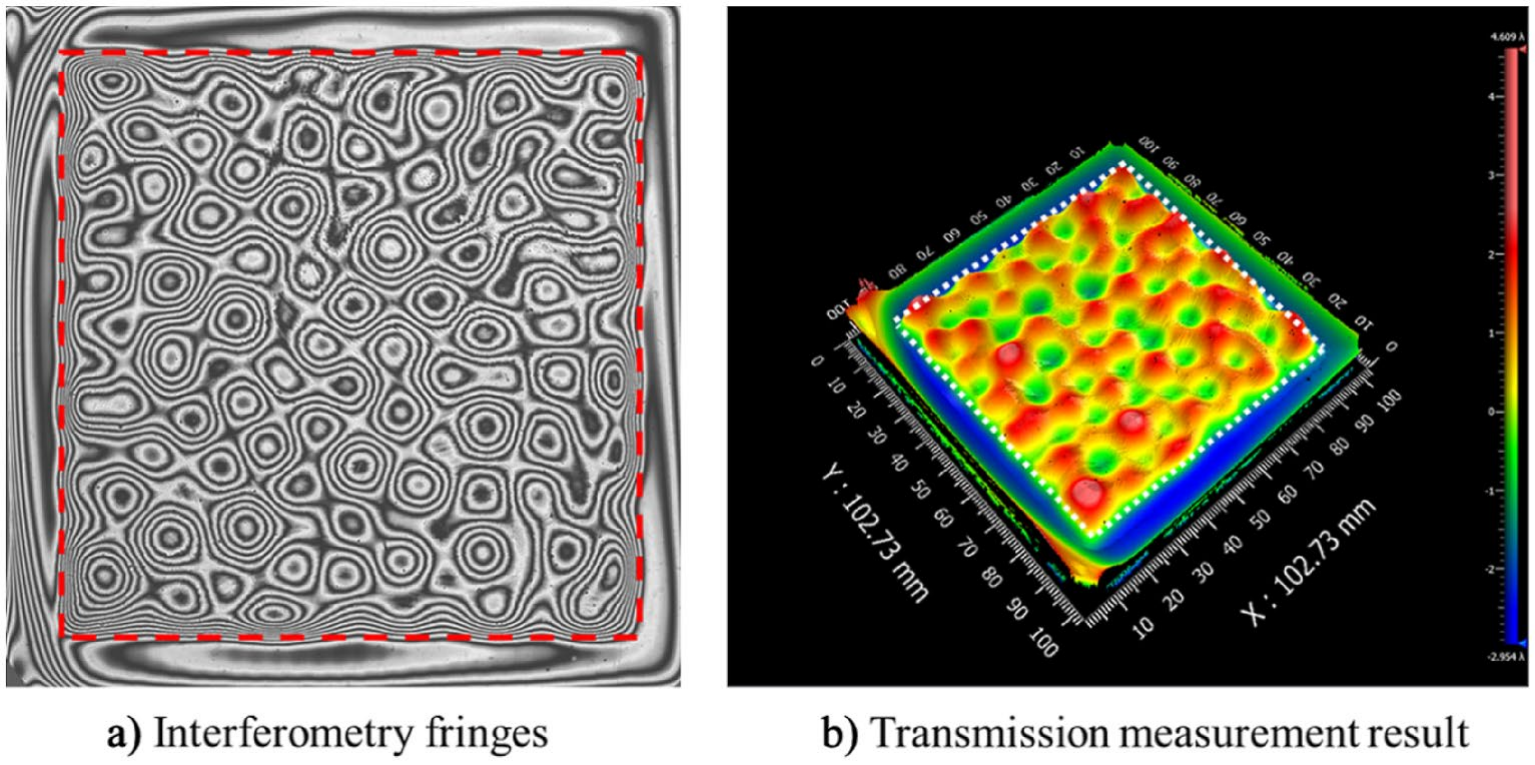
Zygo interferometer transmission measurement.

**Figure 18:**
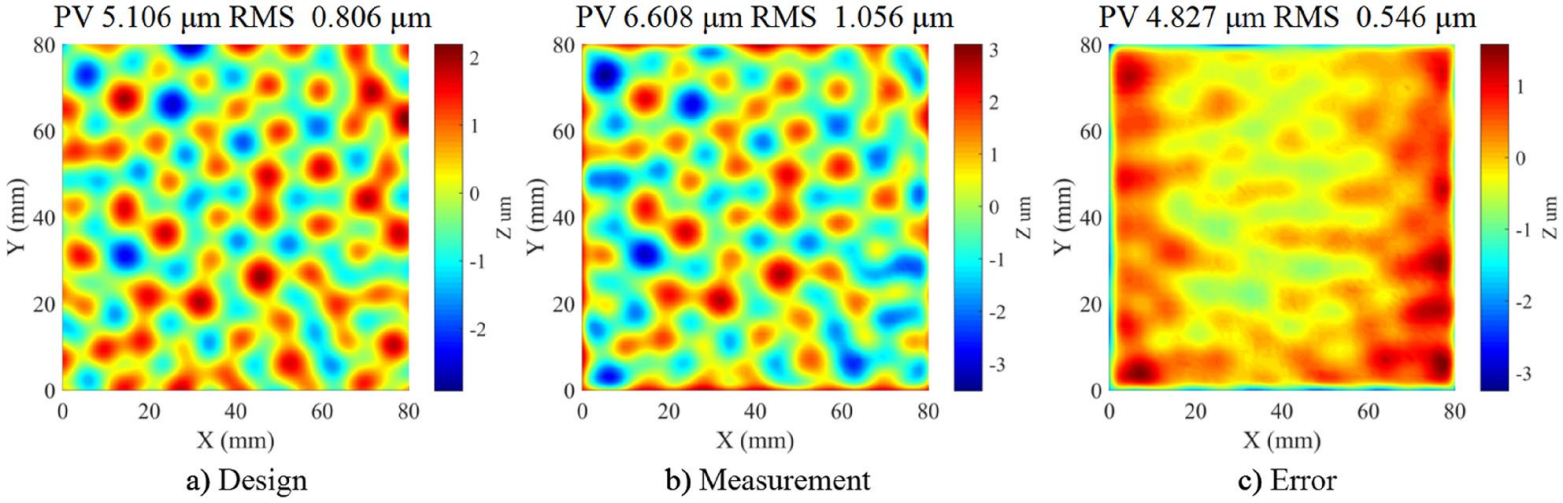
Continuous APPS experiment results of a 3D CPP pattern.

To verify the continuous APPS to fabricate 3D patterns, the experimental structuring of a CPP was performed based on the simulation results as discussed above. The structured CPP sample was then measured in a transmission configuration by Zygo phase shifting interferometer. The result is shown in Fig. 17. The area in the red square is the structured pattern in a single process. The design data and measurement are compared in terms of surface height, respectively shown in Fig. 18a, b. It can be seen that the structuring result is in good agreement with the CPP design. The total machining time is about 100 min. The deviation is mainly located at the surface boarder due to the machining edge effect and the residual initial surface error. The root mean square of surface deviation is 0.546 μm as shown in Fig. 18c. The experimental results indicate the potential of the continuous APPS mode to fabricate 3D complex patterns on fused silica optics.

**Figure 19:**
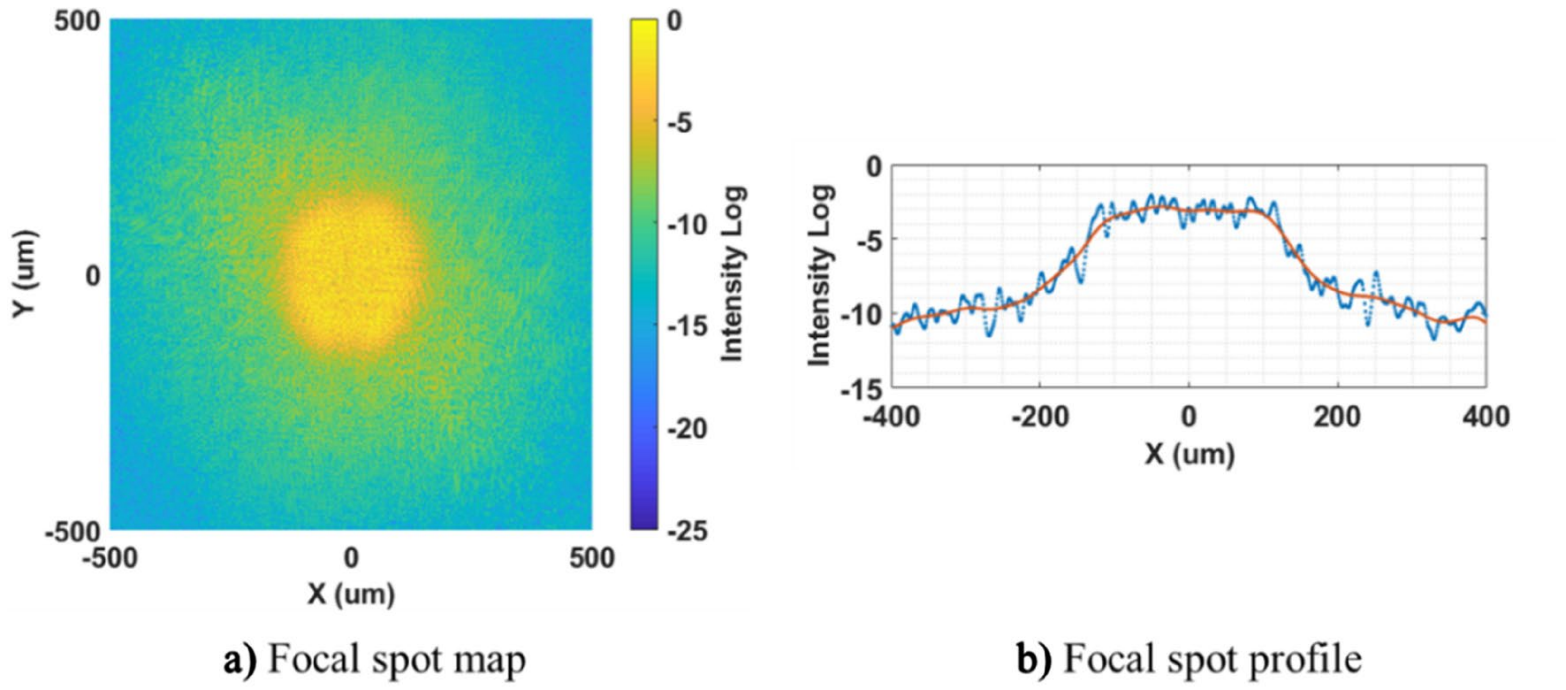
Optical performance evaluation: far field focal spot.

To evaluate the optical performance of the structured CPP pattern, the far field focal spot was obtained at testing wavelength of 351 nm. The focal spot and its cross-section profile are shown in Fig. 19. The focal spot size is about 250 μm. The flat top region at the central spot indicates the CPP pattern generated by APPS can modulate the incident laser to realize beam shaping and thus achieve the uniform illumination of the target surface, which could meet the desired functionality.

## Conclusions

This paper has presented the 2D/3D patterns generation by means of computer-controlled APPS on fused silica optics. The capacitively coupled APPS system with a double-layer plasma torch was developed and its discharge characteristics were investigated. Through multi-physics simulation and process investigation, the stable and controllable Gaussian-shape removal function was achieved. Two different structuring modes, including discrete and continuous APPS, were explored for 2D/3D patterns, and validated through both simulation and practical experiments. A series of structuring experiments have shown that different kinds of 2D patterns (including square lens array, hexagon lens array and groove array) and complex 3D CPP patterns have been successfully fabricated, which validates the effectiveness of the computer-controlled APPS of 2D/3D patterns on fused silica optics.

## Data Availability

The datasets generated during and/or analyzed during the current study are available from the corresponding author on reasonable request.
